# Technologies for well-being: a grand challenge in connected health

**DOI:** 10.3389/fdgth.2024.1503554

**Published:** 2024-10-30

**Authors:** Toshiyo Tamura

**Affiliations:** Institute for Healthcare Robotics, Future Robotics Organization, Waseda University, Tokyo, Japan

**Keywords:** wellbeing, quality of life (QOL), behavior change, IoT, wearable, mental stress, physical stress

## Introduction

In recent years, we have faced various social challenges, such as climate change, declining birth rates, aging populations, and population decline. As COVID-19 causes major changes in the way we work and live, “well-being” is attracting attention as a criterion for “how to change”.

Although no single definition of well-being has been established, the WHO defines that health as not the absence of disease or infirmity, but a state of good health that does not give rise to physical, mental, or social problems. Well-being includes an individual's ability to contribute to society and the world with meaning and purpose, as well as an improved quality of life (1).

We are conducting research and development aimed at realizing a well-being information society where all “people” can enjoy both physical and mental/spiritual health in a world in which the digital and real worlds will continue to merge. Improving well-being through Connected Health means using technology to improve health management and promote overall wellness. Connected Health includes remote monitoring, telemedicine, personal health data, chronic disease management, behavioral health support, etc.

By integrating these Connected Health strategies into daily life, individuals can benefit from more proactive, personalized, and efficient healthcare, ultimately enhancing their overall well-being.

In this challenge, we will present our efforts to realize well-being from different perspectives, including as people and society, as well as physical, mental, and spiritual health.

## Physical and mental well-being

A basic element of well-being is physical well-being. It is important to be healthy and to maintain good physical and mental health, and on this basis, values (symbiosis, empathy, sustainability, happiness, and prosperity) that enhance an individual's quality of life (QOL) will spread. However, humanity is currently experiencing a pandemic of a new type of coronavirus infection and the situation can be said to be one in which physical and mental well-being has been significantly reduced worldwide.

Physical well-being is a basic element of wellness. It is believed that it is important to maintain good physical and mental health, and that good health can serve as a basis for cultivating values (symbiosis, empathy, sustainability, happiness, and prosperity) that enhance one's quality of life. The medical field is currently undergoing a paradigm shift from symptomatic therapy, which seeks cure after illness, to a world of care aimed at preventing illness in the first place (preventive medicine).

An important perspective on well-being is behavior change, and the EU has been looking for effective behavior change for many years. In particular, the behavior changes facilitated by wearable devices and the Internet of Things are the result of advances in engineering and computer science. For example, much development research has focused on case studies of how wearable-based vital sign monitoring can reflect behavior change, and it highlighted the relevant and urgent research in this important area of understanding human behavior change through technology.

The findings of Del-Valle-Soto et al. demonstrate the effective role of wearable and IoT devices (“connected health devices”) in promoting positive behavior change and improving the well-being of individuals in their social environments, and they also emphasized the need for continued research and development in this area to explore technologies that would help people live healthier and happier lives (2). Connected Health can support both physical and mental health through applications that offer therapy exercises (3, 4), mood tracking (5–7), and stress management techniques (8, 9). Virtual support groups and counseling services can also play a role. In addition, connected health systems can aggregate and analyze personal health data, providing insights tailored to an individual's specific needs. This personalized approach can improve the effectiveness of treatments and lifestyle changes. Many connected health devices offer the ability to set health goals and track progress, which can help individuals stay motivated and make positive lifestyle changes.

These researches emphasize key benefits of connected health devices to improve behavior change, including the potential for self-monitoring, incorporating elements of gamification, supporting steady state through individual data management, and leveraging social influence. Gamification for elderly care has shown promise in influencing intentions to use new technology-based solutions (10). Sestino and D'Angelo found that digital therapies based on gamification either preserved cognitive function or slowed the rate of decline in elderly, thereby increasing their social participation.

In addition, connected health management is also working to realize “bio-digital twin technology,” which enables the collection of a wide variety and large amount of bio-data from everyday life, modeling human physiological functions, and simulating future predictions that capture the characteristics of each individual.

Sensor-based digital twins can assist sensors and connected health devices in automatically performing their monitoring tasks from the smart environment (11). Adibi et al. focused their research on the healthcare endpoints and outcomes of integrating sensor technology, digital health capabilities, and navigation systems in an intelligent environment. By analyzing digital twin applications, they concluded that sensor-based digital twins contribute to the well-being of physicians, providers, and patients.

As mentioned earlier, the medical field is undergoing a paradigm shift from symptomatic treatment to disease prevention (preventive medicine). The use of advanced bio-information processing technology based on the digital mapping of an individual's bio-information will lead to well-being by avoiding unknown disease risks in advance, preventing disease by naturally inducing healthy behaviors and supporting independent living in one's way. For example, electronic health records (EHRs) use health devices connected to machine learning technology to provide pre-registered participants with more secure, real-time, patient-centered records. Data is collected and machine learning algorithms determine status and further detect health abnormalities. Such systems are intended to improve health prediction at institutions for the overall well-being of participants. A current trend is the implementation of predictive medical systems for early diagnosis (12).

On the other hand, during social reforms such as the promotion of remote working, there are reports of increased physical and mental stress caused by working in environments other than the workplace. There is also concern that reduced physical activity and more relaxed lifestyles may lead to the onset or worsening of lifestyle-related diseases. Maintaining good health often requires “persistence” and “effort” to discipline behavior and establish regular lifestyle habits. By accumulating biosensing data on daily activities and analyzing the characteristics of lifestyle habits (diet, exercise, and sleep), which are the basic elements of well-being, on a time axis, a method has been acquired to reveal the biorhythmic characteristics of individuals. Combining this with knowledge from human behavioral psychology and behavioral economics, it is important to establish a mechanism to automatically regulate the rhythms of body and mind by presenting and indexing evidence of spontaneous and effortless changes in healthy behavior and developing feedback methods.

The body clock that generates the human circadian rhythm is regulated by the input of external light and other information to a specific region of the brain (the suprachiasmatic nucleus). Recent studies have shown that understanding circadian rhythms and eating nutritious, well-balanced meals at regular times of the day can lead to a healthier diet. In other words, by incorporating a diet that takes into account the body's internal clock into daily life, it should be easy to develop a healthy lifestyle. Chrononutrition has mainly been tested on nocturnal animals such as mice. Evidence is accumulating on the relationship between human circadian rhythms and healthy eating habits (13, 14).

## Social well-being

Social isolation has become a major issue due to the transformation of communities as a result of population decline. The quality of life of older adults, especially those who are more socially isolated, needs to be improved.

Explore how Connected Health can be used to improve the social well-being of socially isolated individuals, such as the elderly and shut-ins, and the effectiveness of interventions based on its use (15, 16).

Engaging older adults at the community level can provide and intervene with technologies to address social isolation, e.g., behavior tracking, room environment measurement, etc., and promote effective practices to increase digital use self-efficacy in the population's mobility, infrastructure, and environment.

Connected Health improves social well-being by using technology to enhance social interactions, support mental health, and foster community connections. Connected Health improves the use of video calls and online consultations to make healthcare more accessible, especially for those who have mobility issues or live in remote areas (12, 17). This can have the effect of improving access to mental health support and the reduction of social isolation.

Healthcare platforms that connect people with similar health conditions or concerns can provide emotional support and practical advice. These groups can also encourage a sense of community and belonging.

Connected Health creates or joins online communities focused on health and wellness where members can share experiences, tips, and encouragement.

In addition, connected health devices provide an understanding of individual health behaviors and deliver tailored interventions or reminders that encourage social engagement and self-care.

Also, develop personalized health plans that incorporate social activities or community engagement as part of the overall wellness strategy.

Mobile technology applications not only connect older adults living alone with their loved ones but also facilitate healthcare and telemedicine for the older adults keeping them mentally and physically healthy. The use of connected health devices compensates for cognitive, visual, and auditory deficits and increases older adults’ self-efficacy in digital use. We recommend a multifaceted solitary intervention, both technical and non-technical, that provides a variety of devices to enhance older adults’ technical skills and support different types of interactions based on individual preferences.

## Future prospective

Future connected health research involving well-being should allow for exploring the effectiveness of various real-world situations, i.e., health care, rehabilitation, sports, routine wellness monitoring, etc., where the implementation of the system should enhance well-being.

As health devices and AI-based analytics continue to develop rapidly, we must observe their effectiveness and long-term effects through connected health devices that integrate them. Through the development of new connected devices and the use of machine learning and deep learning to identify behavioral changes, and develop disease prevention and prediction algorithms, we can work to improve individual well-being.

With this in mind, we believe that the potential of connected health systems is a growing area of research in the pursuit of physical and mental well-being. Such developments will benefit healthcare systems by enabling them to anticipate health problems and to diagnose, treat, and monitor patients in both outpatient and inpatient settings. As more technology-enabled healthcare services are introduced to enable health systems to provide flexible models of care, traditional medical practices will increasingly be augmented or complemented by connected health devices. However, the introduction of connected health devices into healthcare must be preceded by the reliability and validity of the devices, i.e., whether they meet the criteria for approval as a medical device. Many smartphone-based vital signs monitors lack the accuracy and reliability as medical devices. And they require maintenance and inspection related to supply and use. Management of the acquired data depends on clear and strong codes of practice regarding its confidentiality, privacy, and cybersecurity. There are issues that need to be addressed in future research related to connected health technologies, the health systems that integrate them, and the users of connected health technologies. Specifically, the first would be the standardization of connected health devices. It is necessary to address whether the devices as well as the processing can be designed with standardized protocols to ensure interoperability with international and interstate global health systems.

In addition, there is a centralized, cloud-based approach to data management in connected healthcare, but the cost of maintaining this approach is enormous. Cloud storage also presents data security challenges. Centralized management on servers in the cloud makes it vulnerable to hackers and other attacks, and the facility managing the servers could be hit by a natural disaster. In the worst case, all data could disappear in an instant. This is where the incorporation of blockchain technology can help solve the problems of cloud storage. It is characterized by the fact that data is recorded in a highly tamper-resistant manner. Furthermore, even in the unlikely event of tampering, the fact that the data was tampered with is recorded. By combining blockchain, it is possible to automate a number of processes: connected health devices collect, blockchain accumulates and operates the data, and AI performs the analysis. Therefore, data management must also be considered.

Finally, consumer and clinician acceptance and digital literacy in the context of using connected health devices to improve health care delivery and overall experience also needs to be determined. We believe that addressing these areas for future research will enable a broad spectrum of connected health research and ultimately contribute significantly to reducing healthcare costs, improving patient-centered care, and enhancing the physical and mental well-being of participants.

## Conclusion

While well-being research has been actively discussed mainly in the fields of psychology and sociology, there has been little research on the design of information and communication technologies for well-being. However, with the development of wearable devices and contactless device technology, many devices have emerged that can measure daily behaviors without burdening the person being measured. In the future, it will be necessary to design information and communication technologies that promote the well-being of individuals and society while addressing the research challenges.
